# Factors Associated with Honey Bee Colony Losses: A Mini-Review

**DOI:** 10.3390/vetsci7040166

**Published:** 2020-10-30

**Authors:** Peter Hristov, Rositsa Shumkova, Nadezhda Palova, Boyko Neov

**Affiliations:** 1 Department of Animal Diversity and Resources, Institute of Biodiversity and Ecosystem Research, Bulgarian Academy of Sciences, 1113 Sofia, Bulgaria; boikoneov@gmail.com; 2 Research Centre of Stockbreeding and Agriculture, Agricultural Academy, 4700 Smolyan, Bulgaria; rositsa6z@abv.bg; 3 Scientific Center of Agriculture, Agricultural Academy, 8300 Sredets, Bulgaria; nadejda_palova@abv.bg

**Keywords:** honey bee losses, colony collapse disorder, *Varroa destructor*, viral diseases, nosematosis, negative pressures

## Abstract

The Western honey bee (*Apis mellifera* L., Hymenoptera: Apidae) is a species of crucial economic, agricultural and environmental importance. In the last ten years, some regions of the world have suffered from a significant reduction of honey bee colonies. In fact, honey bee losses are not an unusual phenomenon, but in many countries worldwide there has been a notable decrease in honey bee colonies. The cases in the USA, in many European countries, and in the Middle East have received considerable attention, mostly due to the absence of an easily identifiable cause. It has been difficult to determine the main factors leading to colony losses because of honey bees’ diverse social behavior. Moreover, in their daily routine, they make contact with many agents of the environment and are exposed to a plethora of human activities and their consequences. Nevertheless, various factors have been considered to be contributing to honey bee losses, and recent investigations have established some of the most important ones, in particular, pests and diseases, bee management, including bee keeping practices and breeding, the change in climatic conditions, agricultural practices, and the use of pesticides. The global picture highlights the ectoparasitic mite *Varroa destructor* as a major factor in colony loss. Last but not least, microsporidian parasites, mainly Nosema ceranae, also contribute to the problem. Thus, it is obvious that there are many factors affecting honey bee colony losses globally. Increased monitoring and scientific research should throw new light on the factors involved in recent honey bee colony losses. The present review focuses on the main factors which have been found to have an impact on the increase in honey bee colony losses.

## 1. Introduction

Managed honey bees are the most important pollinators for many crops and wild flowering species. Many countries worldwide, particularly in the Northern hemisphere, rely on the Western honey bee, *Apis mellifera*, for commercial pollination of certain crops, but over the recent years there has been an increase in losses in managed honey bee colonies in some regions of the world. Colony collapse disorder (CCD) has been reported for the first time in 2006 in the USA [1]. Although some bee losses have also been reported in China and Japan, published data from various investigations have shown that honey bee colony numbers have been stable for the past ten years in these regions [2,3]. The global picture has shown that there are no significant honey bee colony losses reported in Africa, Australia and South America. In the Middle East, the high temperatures and droughts in the summer are the main factor leading to colony losses because many plants, which are important sources for bee forage, suffer from heat stress [4]. Another factor aggravating the problem is the lack of comprehensive laws and legislations concerning the importation of bee colonies [5].

Indeed, bee colony losses are not a new phenomenon, and historical records show that extensive losses were not unusual in the past. Whilst recent problems may give the impression that there has been a massive decline, global research on honey bee colonies has shown that numbers actually increased between 1961 and 2007, mostly in Asia (426%), Africa (130%), South America (86%), and Oceania (39%) [6]. The most significant honey bee colony losses take place during overwintering, as shown by comparisons of colonies going into wintering and surviving the winter. The latter is a symptom of CCD, which has appeared in Europe, causing losses of up to 30% in some countries [7,8,9]. It has been difficult to determine a common pattern for the colony losses, but different investigations confirm that it is a phenomenon characteristic of the Western honey bee, while the Asiatic honey bee, present in southern, southeastern, and eastern Asia, appears to be more resistant to various pests and diseases [10].

## 2. Role of Pests and Diseases as Drivers Leading to Honey Bee Colony Losses

To understand the causes underlying the current decrease in honey bee colonies worldwide, it is important to shed light on the key pests and diseases that negatively affect bee health. Honey bees can be affected by various pests and diseases, including mites, different viruses, microsporidia, bacterial infections, and fungi (*Ascosphaera apis*) (Table 1). Due to the burden of infectious diseases and their agents, honey bee colonies may manifest significant weakness or even death. Only recently have scientists come to understand better the impact of the development and interactions of these pests and diseases.

### 2.1. Parasitic Mites

Honey bee hives can be a suitable habitat for various mites (Acari), including nonparasitic, omnivorous, pollen-feeding species, and parasites. Out of the different mite species associated with honey bees, *Varroa destructor*, *Acarapis woodi*, *Varroa jacobsoni* and *Tropilaelaps clareae* are economically significant pests of honey bees, and their infestation may lead to the destruction of the beekeeping industry in many cases [31,32]. *Varroa destructor* is the most serious pest of honey bee colonies around the world, as it is an obligate parasite which is able to attack different developmental stages and castes of *A. mellifera* [33]. It is interesting to note that Varroa mites have been established in New Zealand since 2000, but as of yet, Australia is still Varroa-free [34]. Additionally, in Africa, African honey bees seem to survive despite the presence of *Varoa destructor*, as do the Africanized honey bees in South America [35]. It is well known that the ectoparasitic mite *Varroa destructor* switched hosts from Eastern honey bees (*Apis cerana*) to Western honey bees (*Apis mellifera*) [36]. Thus, the Western honey bee has shown more susceptibility than *Apis cerana*. This increased resistance of the Africanized honey bees against *V. destructor* may be explained with their more aggressive behavior than the Western honey bee [37,38]. The association of *V. destructor* with the Western honey bee has led to a significant reduction of honey bee colonies.

*V. destructor* feeds on the fat bodies from adult bees, but not with hemolymph as previously believed [39]. For drones, it has been demonstrated that reduced weight due to *V. destructor* has resulted in decreased flight performance and sperm production [40]. There have also been reports of impaired orientation and homing ability of foraging bees infested with mite parasites, where the affected bees needed more time to return to the colony or did not return at all [41].

Therefore, a moderate Varroa infestation is considered to be one of the major factors leading to reduction of honey bee colonies, due to its influence on reproductive capacity and the general fitness of the colony. Moderate-to-severe infestations have been observed, especially during the autumn, when the mite population increases whereas the host population decreases [42]. The damage threshold is not correlated exactly with the number of mites per colony but is highly variable, depending on the bee and brood population, the season and its role as a very efficient vector of several honey bee-associated viruses [43].

Typical control of *V. destructor* involves the use of strips treated with fluvalinate, a pyrethroid, which are placed in the hive during times of no honey production. Intensive use of these strips has resulted in resistance to pyrethroid insecticides in some parts of Europe [44], the United States [45], Israel [46], and Mexico [47]. The spread of pyrethroid resistance in Europe seems to follow the initial spread of the mite according to bee movement, which suggests that resistance evolved once and spread thereafter [48]. Coumaphos, an organophosphate insecticide, was soon introduced for emergency use after control problems with fluvalinate. However, resistance to coumaphos is already present in Florida, USA [49] and northern Italy [50]. In Minnesota, USA [51] and in Mexico [47], there have been reports of resistance to both pyrethroids and amitraz, an amidine.

Thus, the increased resistance of *Varroa dectructor* against various insecticides creates a precondition for additional difficulty in combating mites and seeking alternative approaches.

### 2.2. Honey Bee-Associated Viruses

About 24 honey bee-associated viruses have been identified in the Western honey bee (*Apis mellifera*) [52]. Some of them generally persist in the bee’s body, without causing a disease or manifestation of any clinical signs. In general, virus infestations were not considered to be a significant problem to honey bee health. On the other hand, some viruses are more virulent and infective, and thus may cause a significant loss in honey bee colonies as well as a decline in honey bees’ health and production [53]. Some viruses show pathogenicity only under certain favorable environmental conditions.

Varroa mites *V. destructor* are considered to be the main transmitter of many honey bee viruses: deformed wing virus (DWV); acute bee paralysis virus (ABPV), Kashmir bee virus (KBV), and Israeli acute paralysis virus (IAPV) [39,54]. Furthermore, three viruses in the transmission of which Varroa seems to play no significant role, namely, chronic bee paralysis virus (CBPV), sacbrood virus (SBV), and black queen cell virus (BQCV) are also frequently surveyed [55,56]. This fact allows to us think that Varroa mites alone are not the (only) cause of honey bee losses. The negative influence of *V. destructor* results from its role as a viral reservoir and a transmitter of some honey bee-associated viruses [33]; the mite promotes replication of honey bee viruses like DWV [57]. Due to its feeding behavior, the Varroa mite injects directly viruses in the hemolymph, which has been associated with oral or sexual transmission of these viruses [58].

### 2.3. Microsporidia

Microsporidia are fungal, obligate intracellular parasites, infectious to honey bees. Microsporidia are possibly the smallest single-cell organisms which have a true nucleus. The genus *Nosema* is a parasitic fungus infecting insects such as honey bees, bumble bees and silkworms. Until now, only two species of microsporidia, namely, *Nosema ceranae* and *Nosema apis*, have been reported to parasitize on adult honey bees [59]. In 2017, a new species of Nosema, named *Nosema neumanni*, in honey bees from Uganda was reported [22]. It has been established that *N. apis* is specific for the Western honey bee, *Apis mellifera* L., whilst the Asiatic bee, *Apis cerana,* harbors *N. ceranae* [60]. For a long time, it was believed that *N. ceranae* and *N. apis* were species-specific. Since the beginning of this millennium (mainly post 2003), many investigations have revealed that *N. ceranae* has switched hosts and has become the dominant species in many countries [61,62,63,64,65]. Thus, it has been suggested that *N. ceranae* is possibly more virulent than *N. apis*.

It has been well documented that microsporidia invade the midgut epithelial cells of worker bees, queens and drones [66]. *Nosema* has adverse effects on the bee colony. The negative effect of nosemosis at the colony level is connected with the productivity and survival of honey bee colonies, including adult bee longevity, queen bees, brood rearing, bee biochemistry, pollen collection and other bee behaviors [67].

In contrast to *N. apis*, which rarely leads to the death of a diseased colony, since its emergence as a novel pathogen of the Western honey bee *A. mellifera*, *N. ceranae* has been generally associated with heavily diseased honey bee colonies [21]. Considering *N. ceranae* as a potential factor in CCD, we may summarize that almost any given disease organism has to persist over time (i.e., there has to be an increase in larval/adult incidence of infection) before causing colony mortality, and generally, *N. ceranae* acts simultaneously with other pathogens. For example, *N. ceranae* has been reported to be persistent for over an 18-month period in the colony before causing colony weakness [68]. Therefore, the possibility that *N. ceranae*, alone or in combination with other factors, causes CCD is still left open.

Bicyclohexylammonium fumagillin, an antibiotic isolated from the fungus *Aspergillus fumigatus*, has been the only widely used treatment for nosemosis, or “nosema disease”, in Western honey bees, *Apis mellifera* [69]. The practice of periodic fumagillin treatment leads to decreasing yet nearly constant exposure of multiple generations of bees and pathogens to the drug. Although this practice appears to provide an environment conducive to selection of fumagillin-resistant Nosema strains, *N. apis* does not seem to have developed resistance to the drug. However, studies have shown that *N. ceranae* can reestablish pretreatment prevalence 6 months after treatments are terminated [70]. Research on protein profiles of bees treated with fumagillin has confirmed our hypothesis that fumagillin affects bee physiology at concentrations that no longer suppress *N. ceranae*. Thus, the use of fumagillin may increase the prevalence of *N. ceranae* and may be a factor in the replacement of *N. apis* by *N. ceranae* in US apiaries [71]. Besides having negative effects on host physiology, fumagillin presents some other drawbacks, e.g., increased management costs and risks to human health through honey consumption, as drug residues may persist in the hive [72]. Therefore, new alternatives to hard chemicals for Nosema spp. management are needed and sought.

### 2.4. Small Hive Beetle

The small hive beetle (SHB, *Aethina tumida*) is endemic to sub-Saharan Africa and alien for European honey bee pest [24]. During the last 25 years, SHBs were introduced into many countries worldwide, causing great damage to beekeeping as well as wild honey bee colonies [25]. The damages from SHBs occur when the honey bee population is insufficient to protect the honey combs from the scavenging beetle larvae [26]. Both adult and larval beetles are harmful to honey bee eggs and brood, but the larvae caused major damages as they funnel through comb with stored honey or pollen, damaging or destroying capping and comb [27]. Unlike wax moths, the SHB larvae do not necessarily damage the combs themselves, and do not produce extensive webbing. When large numbers of adult beetles defecate in the honey, they introduce yeasts, causing the honey to ferment and run out of the cells [28]. In this case, the queen bee may cease laying, and the entire colony may abscond. Weak colonies are particularly vulnerable to attack, but even strong colonies can be overwhelmed by large populations of beetles [24].

Beetles can create problems in cases when honey is removed from the hive, but not immediately extracted, i.e., beetles can invade the beekeeping room and quickly ruin a large portion of a honey harvest [29]. Honey contaminated by SHBs often is rejected by honey bees and is entirely unfit for human consumption [30].

### 2.5. Synergistic Effects of Various Diseases and Parasites

The interaction of Nosema spp. and viruses has been reported for bees co-infected with *N. ceranae* and chronic bee paralysis virus (CBPV) or DWV. One study showed that co-infection of bees with *N. ceranae* and CBPV led to increased replication of CBPV but did not result in mortality [18]. Costa et al. [73] found a significant negative correlation between N. ceranae spore load and DWV titer in midgut tissues of workers.

An interesting study has evaluated co-parasitism with Varroa (*Varroa destructor*) and Nosema (*Nosema ceranae* and *Nosema apis*) on honey bees (*Apis mellifera* L.) which have different defense levels [74]. The obtained results have shown that high-mite-mortality-rate (high-MMR) bees in the Nosema (−) group exhibited greater reductions in mean abundance of mites over time in comparison with low-mite-mortality-rate (low-MMR) bees, when inoculated with additional mites. However, high-MMR bees did not manage to reduce mite load as well as in the Nosema (−) group when fed with Nosema spores. As a whole, the study demonstrated differential mite mortality rates among different genotypes of bees, and also showed that Nosema infection inhibited the success of Varroa removal in high-MMR bees [74].

Other authors found considerable colony level variation regarding infection levels, and identified subtle differences between the microbiota of colonies with high infection levels and those with low infection levels [75]. Two exact sequence variants of Gilliamella, a core gut symbiont, previously associated with gut dysbiosis, were notably more abundant in bees from colonies with high Nosema loads in comparison with those with low Nosema loads.

An interesting study has been conducted to examine the association between *Varroa destructor* and DWV spreading from it in infected honey bees [76]. The study is a new approach to clarifying pathogen–parasite interactions in bees. The results obtained show that the experimental removal of increasing volumes of hemolymph from individual bees increases viral densities. The conducted research clearly demonstrates the defensive role of hemolymph in the bees and confirms that hemolymph has not only a bactericidal mode of action but also affects the regulation on viral replication in infected bees, which has been shown for the first time. The pathogen–parasite relationships observed in honey bees confirm their negative impact on colony losses [76].

Another interesting research study is related to a not so common symbiosis in bees [77]. The Varroa mite and DWV act in mutualistic symbiosis in infected honey bees, i.e., *Varroa destructor* vectored DWV, whereas the DWV-induced immunosuppression in honey bees is mediated by NF-κB signaling pathway, which facilitates mite feeding and reproduction. The decrease in the immune response of bees due to this symbiosis is another reason that leads to a significant weakening of bee colonies [77].

Other examples may be given about synergistic effects of various pathogens in honey bees, but we may conclude that they all lead to honey bee colony losses to a greater or lesser extent.

## 3. Anthropogenic Direct Drivers Associated with Honey Bee Colony Decline

In addition to different pest and diseases as direct natural drivers, there are many other drivers named anthropogenic, that lead to colony losses [78,79]. In many cases it is the interaction of these factors that leads to morbidity and mortality, and colony losses (Table 2). In this review, we will focus on the factors that are considered the most important.

### 3.1. Pesticides

In recent decades, beekeepers have begun to use agrochemical pesticides not only for many crops, but in forests and other environments for the control of insect pests [106,107].

Pesticides are toxic chemicals with a specific mode of action, most often affecting a specific metabolic pathway in an organism [108]. Pesticides are applied via different pathways over the crop—sprays, seed coatings etc. [109]. The exposure of bees to pesticides is through ingestion of residues found in the pollen and nectar of contaminated plants (crop plants or the weeds around the fields) [110]. Insecticides pose the greatest danger to bees [80]. For this reason, beekeepers should be careful when using them near hives. In many countries, it is customary to inform beekeepers before treating crops in order to avoid their toxic effects on bees. These generally accepted rules are not observed in every country, which leads to mass mortality in bees and even the destruction of entire apiaries.

In recent years, the application of a new generation of pesticides—neonicotinoids—has been widely discussed among the scientific and beekeeping communities. They are used worldwide and are widely used for plant protection (crops, vegetables, fruits), veterinary products, and biocides to invertebrate pest control in fish farming [109]. Neonicotinoids represent a neurotoxic compound and their impact is more noticeable in insects than in mammals [111]. Their neurotoxic action is expressed as an agonist at nicotinic acetylcholine receptors (nAChRs) on the postsynaptic membrane, which plays a role in many cognitive processes [112].

One of the main marketed neonicotinoids is imidacloprid [113]. This neonicotinoid has various negative impacts on different biological processes in honey bees [81,82,83]. A recent study has shown that imidacloprid is involved in the reduction in protein biosynthesis and the decrease in the level of proteolytic activity (proteases) [114]. Taking into account the high protein content in royal jelly, this protein reduction is affected extremely adversely by the diet in larvae and queen, i.e., there is a subsequent decrease in honey bee populations [113]. In addition, it has been suggested that imidacloprid has a negative effect on antioxidant protection in young bees [115] as well as reduction in sperm motility (active mitochondria and sperm viability) [116].

These studies have shown the negative role of neonicotinoids on honey bee health and survival, which raises serious objections to their widespread use in agriculture.

### 3.2. Climate Change

Climate change may alter the synchrony between plant flowering and pollinator flight periods. Phenological mismatches probably contribute to pollinator losses that subsequently disrupt pollination of plants [117]. Climate is a crucial factor determining temperature and humidity. Humidity in hives must be maintained as low as possible, while the temperature of the brood must be maintained at 34 °C, and in winter the core temperature of the hive must not fall below 13 °C [118]. This is very important, and honey bee colonies must have sufficient access to carbohydrates to maintain these temperatures and survive. Extended periods of cold or wet weather or depletion of food sources can also have a negative impact on honey bee colony health. These can restrain flying activity and limit nectar and pollen supplies to the hive. In contrast to low temperature, if the brood temperature increases above 34.5 °C, bees display behavioral differences combined with learning and memory difficulties [119].

The effect of weather on bee colonies as a major factor in CCD has been reported in a survey of honey bee colony losses in the USA [120]. CCD has been linked to changes in bee habitats and malnutrition, both of which are indirectly caused by climate change. Moreover, climate change may allow invasive species to invade bee hives, causing disruption and further decline in bee populations [121,122].

### 3.3. Environmental Pollution

Honey bees interact with the environment especially while collecting pollen and nectar for feeding. Thus, they come into contact with some chemical substances and waste from the environment. In nature, waste and toxic substances (generally industrial gases, exhaust gases from vehicles, pesticides and insecticides) are absorbed and stored by plants. The largest part of air pollution is caused by anthropological factors (urbanization, industrialization, energy generation, mobile sources and other pollutants). One of the most important consequences of air pollution is heavy metal pollution [123,124]. Various heavy metal cations such as cadmium, cobalt, copper, zinc, lead, nickel and mercury are known to adversely affect both pollen (directly) and also the honey bees that feed on it (indirectly) [125,126]. Since bees collect pollen from different kinds of flowers, heavy metals found in large quantities within affected plants cause increased concentration of toxic heavy metals in bees’ bodies and poison the latter. That is why honey bees and honey bee products are used as bioindicators of environmental pollution with heavy metals [123,127,128].

### 3.4. Bee Management

Specific peculiarities of beekeeping can be the direct cause or a supplement to the complex of stressors that can contribute to colony breakdown. These include artificial, unilateral feeding, use of antibiotics, acaricides and insecticides in hives, exposure to adverse temperatures and temperature fluctuations, infections and parasites, overexploitation of bee products, as well as unreliable sources of bees and queens [129,130]. One-sided selection of the honey bee results in genetic erosion in the species population and a lack of resistance to infectious diseases, mites, beekeeping acaricides applied in hives, etc. [130,131].

A pan-European epidemiological study aimed to clarify the role of beekeepers on honey bee losses [132]. The authors concluded that honey bees kept by professional beekeepers never showed signs of disease, unlike apiaries from hobbyist beekeepers that had symptoms of bacterial infection and heavy Varroa infestation. Moreover, they claimed that beekeepers’ experience and apicultural practices are the major drivers of honey bee colony losses. To avoid these shortcomings in beekeeping, it is necessary to conduct trans-national monitoring schemes and keep improving beekeeper training [132].

Artificial feeding of bees is often necessary due to overcrowding in conditions of prolonged cold and rainy weather [133]. Feeding is found to be deficient in areas with intensive agricultural production, where the so-called stress from a monotonous or “monocultural” diet is observed [96]. This refers to the continuous foraging of bees on crops in mass flowering, grown in large fields, such as sunflower or rapeseed, and acacia, where the goal is honey production or just pollination of plants. Other factors related to insufficient nutrition include low-nutrient pollen and nectar, as well as certain plant species, including crops and flowers containing substances which are natural but toxic to bees. This is the case for the amygdalin glycoside found in almond flowers [134,135].

Pollen is one of the most important food sources for the proper development and functioning of honey bee colonies. Protein supplements are usually used in early spring to stimulate feeding and in mid-early autumn in order to successfully overwinter bee colonies. It is interesting to note that the addition of small amounts of pollen into protein supplements increases the resilience of honey bee colonies against DWV and Nosema infection [136]. Moreover, seasonal pollens are very important for honey bee feeding and development during their annual cycle [137]. For this reason, it is necessary to plan the presence of pollen annually, and in case of seasonal deficiency in nature, it is necessary to provide additional feeding to the bee colonies. Honey bee nutrition is also an important factor for increasing the resistance of honey bee colonies against various pathogens [138]. Proper and balanced nutrition throughout the year is a prerequisite for the effective function of the immune system at both the individual and social level. Consistently improving nutrition is a necessary condition for higher sustainability and resistance against pathogens (for example Varroa mites and honey bee-associated viruses) and diseases.

It is well known that, in most countries, large numbers of hives are transported by truck to multiple locations to pollinate seasonal fields and orchards. Transportation exposes colonies to many challenging stressors. The ecological conditions to which a hive is acclimated prior to transportation are often quite different from those of the destination. Bees are moved between locations at highway speeds and deployed in fields and orchards prior to the bloom. After transportation bees are exposed to changes in temperature, day length, and nutrient supplementation, which can result in increased foraging activity and brood production earlier than what would have occurred before relocation and in agricultural environments prior to floral bloom with low availability of resources [139]. Although transportation has been noted as a factor likely contributing to colony loss, the focus has been on changing forage quality and consistency rather than on stress endured during transportation [140]. Transportation stress has received less attention due to the difficulties of collecting data during shipping, yet it needs to be taken into consideration.

During transportation, colonies experience a number of stressors, including confinement, increased variation in temperature, air pressure, and vibration, frequent changes in elevation and latitude. Insufficient ventilation poses a serious risk of mortality due to overheating. Low-temperature stress also affects the bees, though less obviously. Transported colonies may experience extended periods of sub-lethal chill stress and loss of thermoregulation (LT), which affects long-term colony survival without proximate mortality by inducing developmental defects in new brood [141,142].

### 3.5. GMO Crops

Soybean and cotton varieties, followed by corn, with genetically incorporated genes for insecticide synthesis and herbicide tolerance, were first introduced in the USA in 1996. In 2007, 113 million hectares in different parts of the world (EU was among the exceptions) were sown with genetically modified crops [143]. With the expansion of the area planted with these crops, concerns have arisen about the safety of bees and other pollinators. Researchers have conducted a number of studies, involving dozens of plant species carrying Bt genes of *Bacillus thuringiensis* for resistance to insect pests [80,144,145]. It has been observed that there are no direct lethal effects of insect-resistant (IR) crops (e.g., producing *Bacillus thuringiensis* (Bt) toxins) on honey bees or other Hymenoptera, but some sub-lethal effects on honey bee behavior have been determined [139]. The impact of GMOs on honey bee health is contradictory. Some researchers have found that ingestion of high concentrations of Bt-toxins affected honey bee behavior [146], while others did not observe differences in the behavior and learning in honey bees [147]. There was no effect at lower toxin concentrations, such as those found in other transgenic varieties [148]. The presence of toxins in GMOS crops leads to reduced larval survival and body mass, and to increased developmental time in honey bees [149]. On the other hand, the introduction of varieties tolerant to certain herbicides and clean field technology have created conditions for growing maize, sunflower, etc., in the absence of weed vegetation in the crops. Since weeds are an alternative source of foraging, the widespread use of technology is considered to be one of the contributing factors to the starvation of bees—both wild and cultivated.

### 3.6. Interactions between Different Drivers

Except that multiple pressures individually impact the honey bee health, there are many evidences about the potential synergistic or additive effects among some drivers that leading to an overall negative impact on honey bee health and survival [12,150]. The most significant interactions among drivers are pointed out: 1. climate change and land-use—for instance, due to global warming, a pollinator species may migrate to a new geographical area, thus increasing the variety of pollinators of recipient region [97]. In many cases, this migration is unfavorable for the local fauna due to competition for food resources or transfer of various pests and diseases [98]; 2. pathogens and chemicals in the environment—the impact of pathogens and insecticides is another form of synergistic effect between drivers. Many authors have observed increased larval or worker honey bee mortality due to the additive or synergistic interactions between sub-lethal doses of neonicotinoid, infection by the *Nosema ceranae* and (BQCV) [99,100,101]; 3. bee nutrition and stress from disease and pesticides—honey bee colonies need proper and balanced nutrition to maintain their development and reproduction [102]. A large number of direct anthropogenic drivers produce alterations in diversity and may even lead to the extinction of many flowering plants which are the main food sources for honey bees [103,104]. These anthropogenic interventions may lead to malnutrition, i.e., reducing activity of immune system and potentially the function of some important detoxification enzymes; there is an elevated risk of the individual and combined impact of pesticides and pathogens on honey bees [96,97,98,99,100,101,102,103,104,105]. From what has been said so far, it is clear that the interaction between anthropogenic direct and natural direct drivers may represent a serious threat to honey bee health and survival.

## 4. Conclusions

Recent investigations have reported an increase in colony losses in some regions and have stimulated investment in more coordinated monitoring of bees and research on the impact of pests and diseases, bee diversity, bee-keeping practices and bee foraging environments on bee vitality. Factors such as land management and environmental conditions further affect the availability and quality of food sources as well as the conditions in the hive. Effective management of bee colonies under changing situations also depends on beekeeping practices and bee selection. All these diverse factors can affect bees’ vitality and ability to overcome pests and diseases.

## Figures and Tables

**Table 1:** Some honey bee pests and diseases correlated with colony losses.

Type of Pathogen	Kind of Relationship	References
*Varroa destructor*	Ectoparasitic mite	[11,12]
*Acarapis woodi*	Tracheal mite	[13]
*Varroa jacobsoni*	Ectoparasitic mite	[14]
*Tropilaelaps clareae*	Ectoparasitic mite	[15]
Deformed wing virus A	Viral pathogen	[16,17,18,19,20]
Deformed wing virus B (VDV1)		
Acute bee paralysis virus		
Kashmir bee virus		
Israeli acute paralysis virus		
Chronic bee paralysis		
Sacbrood virus		
Black queen cell virus		
*Nosema ceranae*	Intestinal parasites	[21,22]
*Nosema apis*		
*Nosema neumanni*		
*Ascosphaera apis*	Fungal pathogen	[23]
*Aspergillus* spp.		
*Aethina tumida*	Beekeeping pest	[24,25,26,27,28,29,30]

**Table 2:** Environmental factors associated with honey bee colony losses.

Anthropogenic Direct Drivers that Cause Honey Bee Decline	Impact on Honey Bee	References
Pesticides	High rate of mortality, alteration of different biological processes	[80,81,82,83]
Climate change	Alteration of honey bee behavior, physiology and distribution, induced changes in flora for honey bees vitality	[84]
Introduction of alien species	Competition for food resources, decline of indigenous species, alteration of the new habitat	[85,86,87,88]
Genetically Modified Organisms (GMOs) crop	Alteration bees foraging behavior	[89]
Land use and management	Habitat and forage loss, honey bee and wild bee competition	[79,90]
Bee management	Hybridity of honey bees, migratory pollination	[91,92,93,94]
Environmental pollution	Imbalance in homeostasis, weakening of the immune system	[95]
Interactions between drivers	In many cases poorly studied	[96,97,98,99,100,101,102,103,104,105]
